# Reply to Mr. Cuming

**Published:** 1804-07-01

**Authors:** 


### *To the Editors of the Medical and Physical Journal.*

Gentlemen,

Mr. Cuming has, as he is justly entitled to, the best thanks of Academicus for his obliging reply, (p. 518 of your 11th volume, to the case stated, p. 461 of the same.) It was before omitted to be noticed, that about the middle of the day, and before dinner, immediately preceding the first day of manifest illness, that is, on the Monday, and before his dinner, the child had suffered a fall of a very few inches, with his whole weight upon his belly, over the knee or thigh of rather a careless or hasty handler, who had so lifted him for the purpose of wiping the soles of his shoes which he had wetted in the grass. But, as the child did not immediately cry, and his body with instantaneous elasticity of the abdominal parts rebounded upon the knee or lower part of the thigh of the handler, who was standing with the left foot upon a step of twelve inches inches above the right foot; and as also, after being loosed, the child went away, and whined but for a very little while, only small and temporary hurt was believed to have been occasioned to him, rather towards the left part of the abdomen, by the indiscreet lifting; which was therefore forgotten for the time.

Animadversion on the treatment will afford pain to those who have witnessed both the knowledge and the judgment of the practitioner in other cases; but, the apothecary certainly expressed an opinion, that if cold had been the cause of the illness, it would have been indicated by a cough. He also sent the saline mixture on the Wednesday, or, at first, to remove the fever, and not for sickness at the stomach, the appearance of which had been very trifling indeed. The fever seemed to have spent itself in its own violence during Wednesday night, and next morning it was gone, in consequence of the saline mixture, as the apothecary expressed his opinion: but coma appeared to have already ensued, at least as one not of the faculty thought. Great debility and much sleepiness had certainly then already ensued; and the apothecary, on his coming, consented to the physician being called in.

No less splendidly expressed than true is the sentiment which Mr. Cuming has delivered in the last sentence of page 520; and the sentence that immediately succeeds it, and closes his letter, induces me to state, that the head of the child was remarkably large, very thinly covered with hair, the seams imperfectly closed, the occiput extended horizontally, but terminating in a conformation almost perfectly hemispherical.

But notwithstanding the largeness of the head, and the peculiar extension of the occiput, the child's health had always been good, excepting an illness from which an emetic had relieved him about a year before; he was robust, active, and occasionally sprightly; at other times he appeared heavy, which was imputed to the size of his head. Two days previously to his last illness, that is, on the Sunday, he had eaten a small, but of course heavy, mince-pye. At the age of three months he passed an easy vaccination, which at six months more was proved good by variolous inoculation. His temper and manners were uniformly easy, quiet, and engaging.

Further Observations upon the case, either from Mr. Cuming, or from any other gentleman of the Faculty, would be an additional favour conferred upon,

Your's, &c.

*June* 16, 1804. ACADEMICUS.

